# Resilience Training Web App for National Health Service Keyworkers: Pilot Usability Study

**DOI:** 10.2196/51101

**Published:** 2025-01-06

**Authors:** Joanna Burrell, Felicity Baker, Matthew Russell Bennion

**Affiliations:** 1 Department of Psychology, University of Sheffield, Cathedral Court, Sheffield, S1 2LT, United Kingdom, 44 7734959002; 2 Ultimate Resilience Ltd, Nottingham, United Kingdom; 3 Department of Computer Science, The University of Sheffield, Sheffield, United Kingdom; 4 Digital Innovation Unit, NHS Midlands and Lancashire Commissioning Support Unit, Stoke on Trent, United Kingdom

**Keywords:** resilience, workplace stress, National Health Service, NHS keyworker, digital learning, digital health, usability, feasibility, mental health, pilot study, learning, training, exercise, primary care provider, health care professional, occupational health, worker, hospital, emergency, survey, questionnaire, mobile phone

## Abstract

**Background:**

It is well established that frontline health care staff are particularly at risk of stress. Resilience is important to help staff to manage daily challenges and to protect against burnout.

**Objective:**

This study aimed to assess the usability and user perceptions of a resilience training web app developed to support health care keyworkers in understanding their own stress response and to help them put into place strategies to manage stress and to build resilience.

**Methods:**

Nurses (n=7) and other keyworkers (n=1), the target users for the resilience training web app, participated in the usability evaluation. Participants completed a pretraining questionnaire capturing basic demographic information and then used the training before completing a posttraining feedback questionnaire exploring the impact and usability of the web app.

**Results:**

From a sample of 8 keyworkers, 6 (75%) rated their current role as “sometimes” stressful. All 8 (100%) keyworkers found the training easy to understand, and 5 of 7 (71%) agreed that the training increased their understanding of both stress and resilience. Further, 6 of 8 (75%) agreed that the resilience model had helped them to understand what resilience is. Many of the keyworkers (6/8, 75%) agreed that the content was relevant to them. Furthermore, 6 of 8 (75%) agreed that they were likely to act to develop their resilience following completion of the training.

**Conclusions:**

This study tested the usability of a web app for resilience training specifically targeting National Health Service keyworkers. This work preceded a larger scale usability study, and it is hoped this study will help guide other studies to develop similar programs in clinical settings.

## Introduction

Resilience allows individuals to manage everyday challenges and changes. For health care professionals who are working in highly emotive and stressful situations, resilience skills are particularly important [1]. It is well established that frontline staff such as nurses are particularly at risk of stress due to factors such as long shifts, organizational pressures, and the emotional impact of their work [2]. During the COVID-19 pandemic, there were high rates of mental health problems among health care staff. For example, a survey of 255 nurses working with respiratory patients found 21% to be experiencing moderate or severe symptoms of anxiety and 17.2% to be experiencing depression. A total of 18.9% scored low or very low on a measure of resilience [3]. A study of 1106 physicians also reported high levels of anxiety and low levels of resilience during the pandemic [4]. Building emotional resilience is therefore imperative to prevent burnout in health care staff, to keep them healthy both physically and mentally, to improve well-being, and to ensure job retention in the workplace [1].

One way that employers can meet this need is through the provision of resilience training. Key benefits of resilience training include improvements in mental health and well-being, social support, self-efficacy, and coping. Further benefits include improved ability to adapt to pressures and demands in the workplace and other areas of life [5]. In health care settings, nurse resilience interventions have been highlighted as a potential way of enhancing staff coping and well-being, job satisfaction, and retention [2]. Greater nurse resilience has also been associated with better work performance [6].

A constraint of traditional resilience training programs is the time required to attend in person, which can exclude certain staff groups, such as nurses, from participation. Smartphone apps have the potential to offer training in resilience to large numbers of people while overcoming barriers, such as stigma, time, and acceptability, and can be integrated easily into the wider organizational well-being strategy [7-11].

The aim of this study was to evaluate whether health care keyworkers would be willing to carry out resilience training via an online platform specifically designed to enable them to understand their own stress response and put in place strategies to manage stress, build emotional resilience, and maintain well-being. The data collected would generate important information for future implementation, while contributing feedback for a more refined usability study with this population.

## Methods

### Participants

The recruitment process was carried out by the Medical Devices Testing and Evaluation Centre (MD-TEC) team. A study sample was recruited from the University Hospitals Birmingham National Health Service (NHS) Foundation Trust, and participation was incentivized with Continuing Professional Development credits for participation. This study was advertised over the web via the internal trust-wide communications bulletin and targeted emails. There was not an enforced inclusion criterion, but this study requested for participants who were a nurse or health care professional and worked in either the emergency department, intensive care unit, or critical care.

### Ethical Considerations

This study was run as a formative usability study by MD-TEC with human participants. The University of Sheffield Re-Use of Existing Data Questionnaire was completed, and the Psychology Research Ethics Committee deemed this study exempt from ethical approval because the data were fully anonymized. A short self-declaration form was submitted. This application went to the Psychology Departments Ethics Administrator for a final check before a letter of confirmation was issued.

Informed consent was derived through the sharing of a recruitment flyer with potential participants. This explained the research and its function as part of medical device usability testing for further development.

All the data were received fully anonymized from MD-TEC post study. The participants were not personally identifiable by the researchers. Research participants were offered Continuing Professional Development credits for their participation. They self-selected for and undertook the research voluntarily.

The training web app was developed by the third author without funding. The content of the training web app drew on the Skills-Based Model of Personal Resilience [12] and included a selection of evidence-based skills and exercises to regulate distress emotions and build positive emotions, such as slow rhythmic breathing and mindfulness practice. The selection of skills and exercises were chosen for their capacity to provide maximum benefit to participants, calming stress, and facilitating improved coping, in the context of this brief trial.

Bennion et al [8] highlight four key indicators of quality drawn from effective digital psychotherapy approaches. These include clinician involvement, academic involvement, research, or other evidence and use of specific psychological approach or theory. The intervention followed these recommendations, drawing on academic and clinical theory [13] and involving clinicians, academics, and computer scientists in its development to ensure greater quality and effectiveness.

The web-based training was published using Articulate Storyline (Articulate Global, LLC) and accessed via a web browser. It consisted of both written and spoken content on a series of slides, short videos, and experiential exercises which could be moved through at participants’ own pace using “previous” and “next” buttons. The estimated time to complete the training was 20 minutes.

### Pretraining Questionnaire

The pretraining questionnaire captured basic demographic information: gender, age range, job role, current area of work, current band, and years of nursing experience. Current job stress was rated on a 5-point scale (never, hardly ever, occasionally, sometimes, and very). Participants were also asked whether they had heard of or previously undertaken any resilience training.

### Posttraining Feedback Questionnaire

The posttraining feedback questionnaire focused on 6 areas: app design and navigation, app content, app impact, app training exercises, app relevance, and app access. Each question was posed on a Likert scale with five possible answer options to allow the user to respond to each statement on a range from “strongly agree” to “strongly disagree.”

### Procedure

Upon contacting the MD-TEC team to participate, interested individuals were provided the opportunity to ask any questions about participation in this study. If willing to consent, participants were then sent the link and password to access the training. The training and surveys were hosted on the MD-TEC Software Usability Testing Site (MD-TEC), and thus could be completed on any device with internet access. Once logged in on an internet browser, individuals were presented with the precourse survey before completing the full training module.

Once the training module was completed, participants were taken to a landing page and requested to click a link to take them to the feedback survey. They were reminded at this point that no identifiable information would be collected from them. As the surveys were completed anonymously, participants who completed the training and survey were asked to inform the MD-TEC team via email once they had done so. They were then sent a certificate toward Continuing Professional Development for their time contributed to research, which they could add to their personal records. The total time for each participant to complete the training module and feedback survey was approximately 45 minutes.

### Statistical Analysis

This study did not use a specific sample size calculation as it was focused on app usability. It instead aimed to achieve at least 5 participants which is deemed an optimal number to reveal 77% to 85% of problems [14]. Data were analyzed using IBM SPSS (version 26; IBM Corp). The pretraining and posttraining feedback questionnaires were summarized as a mix of continuous variables with medians and categorical ordinal variables with percentages.

## Results

### Participants

The age of participants ranged from 25 to 64 years. The total sample (N=8) was comprised of 8 (100%) females, 7 (87.5%) nurses, and 1 (12.5%) keyworker of other professions. Grades ranged from 5 to 7 with a median of 6 (IQR 5-6). Five of the participant’s had over 15 years of nursing experience. All 8 participants completed baseline measures and posttraining measures.

### Pretraining Questionnaire

#### Sample Overview

Of the 8 participants who completed this study, 3 (37.5%) worked in the hospital’s intensive care unit, 1 (12.5%) worked in the emergency department, and 4 (50%) worked in other undisclosed areas of the hospital.

#### Current Role Stress

Most participants (6/8, 75%) rated their current role stress as “sometimes” stressful, while 1 of 8 (12.5%) said “occasionally” stressful and 1 of 8 (12.5%) said “very” stressful.

#### Awareness and Knowledge of Resilience Training

Most participants (6/8, 75%) had heard of resilience training, and those that had taken part (4/8, 50%) had done so in a face-to-face setting.

### Posttraining Feedback Questionnaire

#### App Design and Navigation

Feedback regarding the design of the training was predominantly positive. All participants found the training easy to navigate, 6 of 8 (75%) deemed the default speed at which the training progressed to be acceptable, and 7 of 8 (97.5%) thought the appearance of the buttons was OK.

#### App Content

Feedback for the content indicated that all participants (8/8, 100%) found the training easy to understand, 6 of 8 (75%) felt there was enough text content, 4 of 8 (50%) felt there was enough spoken content, and 5 of 8 (62.5%) felt there were enough interactive exercises.

#### App Impact

A large number of the participants (5/7, 71%) agreed that the training increased their understanding of both stress and resilience, while 6 of 8 (75%) agreed that the resilience model had helped them to understand what resilience is.

#### App Training Exercises

The training exercise feedback was positive but varied. For the breathing and positive tips exercises, 6 of 8 (75%) participants agreed they were likely to try the exercises again in the future. The mindfulness exercise had 4 of 8 (50%) participants agree they were likely to try the exercise again.

#### App Relevance

There was a high level of agreement that the training was relevant to nurses, with 6 of 8 (75%) participants agreeing that the content was relevant to them.

Furthermore, 6 of 8 (62.5%) participants agreed that they were likely to act to develop their resilience following completion of the training.

#### Access to Training

All the participants indicated a different personal preference to how they would prefer to access the training. Participants felt the package should be made available across all platforms to allow the training to be completed where and when it was most convenient to them. When asked their preferred location of access, 5 of 8 (62.5%) indicated their preference as being “at home.”

## Discussion

### Principal Findings

We explored the perceived usability and feasibility of a resilience training web app created for NHS health care keyworkers. Data collected covered a number of areas: design and navigation, content, impact, and relevance. The results showed that 100% (8/8) of participants found the training easy to understand and agreed that it had increased their understanding of both stress (5/7, 71%) and resilience (6/8, 75%). Three-quarters of participants agreed that the content was relevant to them, and this corresponded with the number of participants rating their current role as “sometimes” stressful. Furthermore, three-quarters of participants agreed that they were likely to take action to develop their resilience following completion of the training. This information was used to inform the design of a larger usability study.

A total of 8 participants were recruited, with 7 being from the target population. All participants completed the process from start to finish. Participants successfully carried out what was required of them based on this study’s protocol, although some participants did not complete all the questions asked on the posttraining questionnaire. There was no indication given as to why this was the case. In a follow up usability study [13] validation checks were put in place within the surveys to stop questions from being missed by mistake.

The findings of this study indicated that participants found the training app design and navigation acceptable and usable. However, the measure used was not a standard model of system usability (eg, International Organization for Standardization, 2018). This study’s design was updated to use two validated measures (the System Usability Scale and the Usability Metric for User Experience) to strengthen the robustness of a follow-up usability study [14]. Adding these two additional validation measures to this study’s design helped to strengthen assessment of the training app’s usability.

Participants indicated that the training was easy to understand and that there was enough text content; however, they also indicated that there was a need for the training to have more spoken and interactive content. This fits with a recent study [15] in which nurses’ interactive behavior was identified as an influencing aspect of nurse satisfaction with online learning. Based on these findings, we recommend the training’s interactive content be revisited in its next design iteration.

Most participants perceived that the training increased their understanding of both stress and resilience and that the resilience model had helped them to understand what resilience is. A more robust method of measurement was required to further explore the impact of the training and this study’s design was updated to incorporate ratings of perceived knowledge regarding stress and resilience. These new scales were used in a follow up usability study [14] and found to increase significantly between pre- and postapp training.

The training exercise feedback was positive but varied. Both the breathing and positive tips exercises were well received, with participants agreeing they were likely to try the exercises again in the future. However, only half of the participants agreed that they would try the mindfulness exercise again. This may have been due to the difficulty in carrying out the exercise in a busy work environment.

Many participants agreed that the training was relevant to them and believed that they were likely to act to develop their resilience following completion of the training.

### Limitations

Recognized limitations of usability studies include that testing is conducted in an artificial situation and personal preferences of the participants are not representative of the wider user population [16]. The digital training app used in our study is an early prototype. This may need multiple design developments to create a smartphone app that can be used to deliver the resilience training. The aim of this formative usability study was to assess the acceptability and user perceptions of the current version of the training program. As such, this study is part of the iterative product development process and is different to a summative usability study, conducted for validation and regulatory purposes [17].

This study had a single-group design and advertised for a specific group; however, anyone employed by the trust who contacted MD-TEC regarding this study could be involved. This was done primarily to allow anyone employed by the trust to gain access to training that could benefit them. Potential participants who were unaware of this clause may have been lost because of this decision. The initial training materials were designed with nurses in mind but were not specifically tailored for the demographic. This may have changed participants’ initial perception of the suitability of the training to them personally. A single-group design can limit the ability to draw definitive conclusions about the effectiveness of the training due to its lack of a control or comparison group [18]. However, since this study was focused on the usability of the training and not the effectiveness, and it was not seeking to make a comparative analysis, a single-group design was appropriate.

This study limited its evaluation to perceived usability, which was not obtained through laboratory-based observations. As such, the positive ratings reported may not be representative of true user experience. A heuristic evaluation of the training to detect usability problems was not carried out, due to pandemic restrictions making this problematic to implement. This study used quantitative scales and measures to collect data but did not use qualitative measures to gain deeper insight into what NHS health care staff felt about the training. A measure of time spent using the training was not collected. This could have also given an indication of acceptability. This study used two single Likert scales to measure perceived increases in knowledge about stress and resilience. Studies have shown that perceptions of learning may not reflect knowledge gains, when compared with evidence of actual learning [19]. A more robust method of measuring knowledge retention would have benefitted this study. This could have been achieved by having a pre- and postquiz based on the content of the training to see what knowledge was retained.

While the majority of participants gave positive responses in the evaluation of this study, the generalizability of these outcomes is limited due to the disproportionate number of female participants and participants from a nursing background. Only 1 (12.5%) participant was from a different professional group. This limits the inferences that can be drawn about usability and acceptability of the training to male participants and those with other keyworker roles. It is recommended that future studies recruit a more representative sample to enhance generalizability of the results.

### Conclusions

Overall, the resilience training module was well received by the participants. The participants felt the package was easy to navigate. There was a high level of agreement that the visual delivery of the training was acceptable, as well as the speed at which this was delivered.

A number of techniques demonstrated during the training were also well received, with 6 of 8 participants agreeing that they would use them in future stressful situations. Mindfulness was the only exercise that received more varied feedback, with half agreeing on its utility in the work environment.

Health care staff participating in this study largely agreed that the training was relevant to their group and that the tone of the delivery was appropriate. No clear preference regarding how to access the training was identified, highlighting the need for accessibility via computer, tablet, and smartphone. Participants expressed a wish to to access the training when they have a moment of need and the opportunity in their busy working day.

### Future Directions

As one of the first NHS web-based resilience programs to be tested, this first usability study aimed to understand whether web-based training for resilience is deemed usable and acceptable by health care staff. The results of this study will be used to expand and build upon the initial prototype to make a more interaction enriched version of the training.

This study also provided an understanding of the program’s limitations and highlighted some aspects which require further adaptation for delivery via a new medium. Future research would aim to evaluate the impact of including greater interactivity on engagement and learning. It would also aim to extend the accessibility and acceptability of the program to a wider audience by developing an effective prototype for a smartphone app.

This study was run externally by MD-TEC, who had their own processes for running usability studies of this nature. This study’s design covered some of the key factors required for an effective online survey, but it could have been further improved by seeking acknowledgment with MD-TEC regarding the CHERRIES (Checklist for Reporting Results of Internet E-Surveys) checklist [20].

It is clear from the results that there is a need for future research to evaluate how skills-based learning using web-based training impacts long term resilience. A larger scale study would allow for more in-depth investigation of the impact of such training on participants’ levels of stress and resilience as well as their perspectives on acceptability.

Given the diversity of NHS staff, it will be important for any future study to gather a wide set of demographic information to investigate acceptability and generalizability across diverse populations. With increasing awareness (ie, gained through the COVID-19 pandemic) of the pressures faced by all NHS staff, across a breadth of ethnic and socioeconomic groups, a larger scale study would allow for a wider inclusion criterion covering all NHS staff groups.

## Abbreviations

CHERRIES: Checklist for Reporting Results of Internet E-Surveys
MD-TEC: Medical Devices Testing and Evaluation Centre
NHS: National Health Service
